# Research to stop tobacco deaths

**DOI:** 10.1186/1744-8603-10-39

**Published:** 2014-05-21

**Authors:** Derek Yach, Angela Pratt, Thomas J Glynn, K Srinath Reddy

**Affiliations:** 1 The Vitality Group, New York, New York, USA; 2 The Vitality Institute, New York, New York, USA; 3 Tobacco Free Initiative, WHO, Beijing, China; 4 Cancer Science and Trends, American Cancer Society, New York, New York, USA; 5 Public Health Foundation of India, New Delhi, India; 6 World Heart Federation, Geneva, Switzerland

**Keywords:** Tobacco control, WHO Framework Convention on Tobacco Control (FCTC), Low- and middle-income countries, Research

## Abstract

In 2003, governments adopted the Framework Convention on Tobacco Control, the world’s first global health treaty. In the decade since the treaty was adopted by 178 member states of the World Health Organization, there have been substantial achievements in reducing tobacco use around the world. Research and evidence on the impact of interventions and policies have helped drive this policy progress. An increased and sustained focus on research is needed in the future to ensure that the gains of the global tobacco control movement are maintained, particularly in low- and middle-income countries, which are affected most strongly by the tobacco epidemic. In addition to current priorities, greater attention is needed to research related to trade agreements, prevention among girls, and the appropriate response to nicotine-based noncombustibles (including e-cigarettes).

## Background

The background to this article is a workshop held at the National Institutes of Health (NIH) in June 2013 to review progress and achievements in global tobacco control research in low- and middle-income countries (LMICs) over the last decade. The commentary is informed by some of the key themes that emerged from this discussion complemented by our views about future priorities for tobacco control research.

In 2003, governments adopted the World Health Organization’s Framework Convention on Tobacco Control (WHO FCTC) [1] to “protect present and future generations from the devastating consequences of tobacco consumption and exposure” [2]. At that time, up to 1 billion tobacco deaths were projected to occur in the 21^st^ century absent sustained action to reduce rates of tobacco use; approximately 80% of these deaths will occur in LMICs. The FCTC’s development was based on the best available epidemiologic and economic evidence on the impact of interventions and policies [3-5], along with insights drawn from analyses of tobacco industry behavior in thwarting past efforts to advance tobacco control [6,7].

The FCTC specifically highlighted ongoing research, surveillance, and the exchange of information as being critical to tobacco control. For example, the Convention advised countries to establish national systems for the epidemiologic surveillance of tobacco consumption and related social, economic, and health indicators. Countries were also advised to cooperate with competent international and regional intergovernmental organizations and other bodies, including governmental and nongovernmental agencies, in regional and global tobacco surveillance and exchange of information on these indicators. The FCTC also emphasized the exchange of publicly available scientific, technical, socioeconomic, commercial, and legal information, as well as information regarding practices of the tobacco industry and the cultivation of tobacco. This exchange should take into account and address the special needs of developing countries and those with economies in transition [8]. Specific research priorities have not been developed by the Conference of the Parties to the FCTC. Rather, research bodies have set priorities based on strengthening the most effective FCTC interventions, many of which are now included under the MPOWER program (Table 1) [9], and by drawing on experts’ opinions [10-12].

**Table 1:** **The six components of MPOWER**[9]

**1.**	● Monitor tobacco use and prevention policies
**2.**	● Protect people from tobacco smoke
**3.**	● Offer help to quit tobacco use
**4.**	● Warn about the dangers of tobacco
**5.**	● Enforce bans on tobacco advertising, promotion, and sponsorship
**6.**	● Raise taxes on tobacco

In this article, we reflect on the role of research in advancing tobacco control, both in the successes to date of the global tobacco control movement and, in light of these, the research priorities that are needed to underpin future progress in tobacco control. We present these views mindful of several negative trends in tobacco use prevalence and of the need for, and new opportunities to develop, innovative approaches to implementing tobacco control policies in many LMICs.

### Current situation

A decade after adoption of the FCTC, global tobacco control is at a critical juncture. Smoking prevalence in many high-income countries is decreasing, but in most LMICs this is not the case [12]. In fact, the global burden of disease attributable to tobacco smoking has not changed significantly, because the decreases in high-income regions are offset by increases in low-income regions, such as Southeast Asia and East and South Asia [13].

Funding for international tobacco control research initially came from Canada’s International Development Research Centre, which initiated Research for International Tobacco Control to address the economic and development aspects of tobacco control [14]. The most significant globally available investment came from the NIH’s Fogarty Center for International Health [15]. Additionally, for more than a decade, the U.S. Centers for Disease Control and Prevention has funded and managed the Global Youth Tobacco Surveys and Global Adult Tobacco Surveys in more than180 countries [16]. Although the United States has been a supporter of research and surveillance along with only a few major countries (including Russia and Indonesia), it has yet to adopt the FCTC.

Around the time the FCTC was adopted by WHO and at the request of WHO, there were notable increased investments in global tobacco control research and surveillance. These included support through the Centers for Disease Control and Prevention for tobacco surveillance (initially focused on youth and later expanded with the support of Bloomberg Philanthropies and the Bill and Melinda Gates Foundation to include adults) and through the NIH Fogarty International Center’s International Tobacco and Health Research and Capacity Building (TOBAC) program, which was initiated in partnership with the National Cancer Institute (NCI) and the National Institute on Drug Abuse to fund tobacco control research and capacity building in LMICs.

### Surveillance issues

Over the last decade, the prevalence of tobacco use has declined by 10% in Organization for Economic Cooperation and Development countries and increased by 18% in LMICs [17]. By 2010, smoking had become one of the leading risk factors for disease burden in many low- and middle-income regions, including Southeast Asia and East and South Asia [13,17].

From 1990 to 2010, smoking increased from the fifth to the third leading cause of disability-adjusted life years in developing countries [18]. Media interest (as measured through Google trends) and some Organization for Economic Cooperation and Development countries’ investments in tobacco control in general and research in particular have dwindled, but, against this harsh reality, there have been policy research and implementation progress that could reverse the negative trends.

Progress on expanding the reach and frequency of surveillance has been impressive, with more than 180 countries now covered by the Global Youth Tobacco Survey [16], 80 of which have carried out at least 3 rounds of surveys, and 32 countries now covered by the Global Adult Tobacco Survey [17], of which 19 have completed the survey. The WHO STEPwise approach to chronic disease risk factor surveillance has also been widely adopted in LMICs [19].

Basic epidemiologic surveillance data remain the cornerstone of assessing progress and mobilizing action in every country. There is a continued need to support LMICs in strengthening both their national surveillance capacity and their capacity to ensure that data are used more widely to assess the impact of policies and make them more effective. A shift in how health departments use youth tobacco survey data is needed. They could learn from the high degree of urgency and action that is driven by infectious disease surveillance data.

Tobacco control is a new and still developing academic discipline in most LMICs, with the majority of researchers having less than six years of experience conducting tobacco control research, indicating that support for tobacco control education and between-country collaboration is vital to progress [11]. The International Tobacco Control (ITC) Project [20] is an example of this approach. It measures the psychosocial and behavioral impact of key national-level policies of the FCTC. The ITC Project includes international health organizations and policymakers in more than 20 countries inhabited by more than 50% of the world’s population, 60% of the world’s smokers, and 70% of the world’s tobacco users. In each country, the ITC Project is conducting prospective cohort surveys to assess the impact and identify the determinants of effective tobacco control policies on smoke-free legislation, health warning labels, pricing and taxation, cessation, advertising/promotion, and communication/education. ITC has presented findings directly contradicting misconceptions and disinformation from the tobacco industry in their fight against effective tobacco control policies and has initiated an eight-year cohort survey of more than 6,000 adult smokers to guide effective policies in Canada, the United States, the United Kingdom, and Australia, including evaluation of new national policies enacted under the U.S. Food and Drug Administration’s (FDA’s) Family Smoking Prevention and Tobacco Control Act [21].

### Research and capacity building

The importance of investing in research to address global tobacco control effectively cannot be overstated. Put simply, the WHO FCTC is an evidence-based document: there is a need to ensure that the evidence base which supports its continued implementation is up to date, robust, and responsive to new and emerging challenges and issues.

For example, there is now compelling evidence of the impact and high value of the $40 million investment the NIH has made in tobacco control research and capacity development through the TOBAC program. This initiative includes the development of new research insights and knowledge, reflected in 405 peer-reviewed publications; the training of 3,519 researchers and policy makers; 34 grants awarded for research and capacity building in more than 30 countries, with support for new regional and global networks of researchers; and, just as importantly, evidence of the cumulative impact of this investment on informing new and effective tobacco control policies. The program invests in empiric research and capacity building simultaneously, awarding funds for five-year periods to allow researchers to build strong regional relationships and develop trust in partnering countries.

The majority of the research projects funded have been conducted in countries in East Asia, the Pacific, South Asia, Latin America, and the Caribbean. TOBAC has stimulated development of a young cadre of tobacco control researchers across the world who have started to impact the course, content, and focus of national tobacco control policies. TOBAC-funded scientists from India, Argentina, South Africa, and Uruguay have all played important roles in FCTC delegations. From 2002 through 2012, the initiative’s $40 million dollar investment has generated powerful outcomes. For example, in Hungary, a country with some of the highest smoking rates in Europe, TOBAC-funded researchers demonstrated taxes to be an effective policy intervention for reducing smoking prevalence. The findings were presented to legal and public health officers in the local governments, and, consequently, tobacco sales taxes were increased nine times in the next four years. In addition, after engagement with local researchers trained in the TOBAC program, the State Secretary’s cabinet passed national clean air laws that protect nonsmokers in public places [15]. While Hungary held the leading smoking rates in Europe, there were 301 million current smokers in China in 2010 [22]_._ China is currently the largest manufacturer and consumer of tobacco in the world, signaling an urgent need for tobacco control research in this population [23].

It is critical to maintain and expand support for this type of research at country level by governments, funding agencies, and other stakeholders to address emerging global research needs and opportunities. Recent reviews have highlighted the importance of investing in country-specific surveillance and research [24], which can be used to stimulate local political action and support for tobacco control in otherwise crowded policy arenas. Operational research to support expedited implementation and maximum impact of each of the major FCTC articles is critical. This is especially true for those that underpin sustained increases in tobacco product prices; guide development of marketing regulations and tackle brand visibility and the explosion in social media and internet marketing more effectively; stimulate implementation of smoke-free policies, including in homes; integrate tobacco control into major disease management programs such as for cardiovascular disease, cancers, tuberculosis, and maternal and child health; and inform development of trade agreements.

Country-specific research also needs to be complemented by global research focused on emerging technologies and trends. These include innovations such as social media and smartphones, all of which have the potential to be tools for tobacco control, although they also are also being used by the tobacco industry to promote their products. Smartphones can be used to enhance cessation effectiveness and long-term maintenance, especially if combined with behavioral economic measures [25]. For example, the NCI has launched SmokefreeTXT, a free text message smoking-cessation service, which provides around-the-clock encouragement, advice, and tips to teens trying to quit smoking. In addition, the NCI has developed an interactive text messaging library and delivery algorithm for adults in the United States who wish to quit smoking. Similarly, the WHO has launched the mHealth for NCDs program, designed to leverage mobile technology to improve prevention and management of noncommunicable diseases. Mobile phones can also be used in novel ways to stimulate citizen involvement in enforcing tobacco regulations, especially in settings where enforcement capacity is weak. However, smartphones, social media, and gaming also provide new ways of personalizing the marketing of tobacco products even as smoking in movies is addressed.

Innovation also supports growth and continued profitability of the tobacco industry, yet that is rarely studied on a systematic basis and with the intent of adjusting policies to changes in industry tactics and dynamics. We believe that the development of a means of proactively monitoring tobacco industry actions that is linked to high-profile reporting is consistent with FCTC article 5.3. Without such research, the FCTC may well have lacked many provisions.

### Emerging issues

Three other trends demand urgent and substantial research: the increase in tobacco use among girls and women, the rapid emergence of electronic cigarettes (e-cigarettes), alternative nicotine-delivery mechanisms; and the emergence of trade law challenges [26-28].

The FCTC does not explicitly refer to trade policy. However, a preambular line designed to give priority to their right to protect public health was included to signal the importance of tobacco control in all aspects of development [1]. In 2003, World Trade Organization Director-General Supachai Panitchpakdi publicly congratulated the WHO on FCTC ratification, stating, “When dealing with the pressing problems of our age, whether they relate to improving health standards or eradicating poverty, there can be no doubt that the nations of the world must work together. A multilateral approach to problem solving offers all of us the best hope for a better world” [29]. In concert with this statement, the FCTC adoption provides a rationale for having the evidence-based interventions in the FCTC take precedence over legal challenges to their use. Such disputes are currently materializing with respect to Australia’s law to introduce plain packaging of tobacco products, and Uruguay’s introduction of stronger graphic health warnings – both in the WTO and through dispute resolution mechanisms under a series of bilateral investor-state agreements [30-32]. Research on trade, international, mercantile, and domestic law must be made a priority if the integrity and intent of the FCTC to “protect public health” is to be maintained.

The ratio of smoking among boys to that among girls has narrowed and in some cases reversed compared with the ratio for their parents’ generation in all countries for which there are data available [12,16,17]. This portends a massive increase in tobacco deaths among women decades from now. Deeper insights into the driving forces behind these epidemiologic trends are urgently required if more effective policies are to be developed. One reason may relate to use of the pack as a marketing tool (especially in places where there are restrictions on traditional or direct advertising, promotion and sponsorship) – for example, the globalization of “Slim” brands based on Virginia Slims, developed more than four decades ago – a trend which is now increasingly visible in many emerging markets. Novel use of the pack in marketing may play a more important role in increases in smoking among women than previously understood. The FCTC acknowledges the need “to take measures to address gender-specific risks when developing tobacco control strategies (Article 4)” [33], as well as to restrict marketing through the pack itself (Article 11) [34].

The alarming increase in tobacco use among girls demands both political action and focused research [35-37]. In many countries where tobacco use among women is still relatively low, the potential to shape its status as the desired social norm still exists. In countries where rates have increased, the voice and visibility of women in leadership positions in business, politics, sports, fashion, and entertainment in support of tobacco control need to be stimulated. Despite the FCTC’s declaration of commitment to addressing gender disparities, there is a void in research on the drivers of tobacco use among girls beyond the globalization of thin cigarettes and the empowerment of women. There is a need to engage institutions such as the United Nations Entity for Gender Equality and the Empowerment of Women, in research and action which on increasing rates of tobacco use among girls and women in the context of the Convention on the Elimination of Discrimination against Women. Similarly, the FCTC preamble on “the Convention on the Rights of the Child” provides that state parties to that convention “recognize the right of every child to the enjoyment of the highest attainable standards of health” [1], and thus there is a need for the tobacco control community to strengthen collaboration with the United National Children’s Emergency Fund in light of mounting evidence linking maternal tobacco use to low birthweight and other adverse childhood health outcomes. Failure will condemn millions of women and children to die and suffer in the next few decades.

There is a need for well-designed studies on the safety, efficacy, and impact of use (including long-term use) of new nicotine-delivery systems, including e-cigarettes. Sound policy must be based on sound science. At this stage, despite the rapid emergence of a multibillion dollar new revenue category [38], the evidence required to establish sound policy is still evolving.

The potential exists for e-cigarettes to become a useful technology capable of substantially reducing the harm caused by tobacco. However, there are concerns that it could undermine the successes of tobacco control policies to date (in particular with respect to denormalization of smoking and decreasing youth smoking), and the evidence on the health impact of e-cigarette use including over the long term is still relatively sparse. In the United States, Congress has released findings that some companies are boosting advertising aimed at youth [39], and the FDA has recently announced draft regulations on e-cigarettes. Other countries and jurisdictions are also grappling with the question of how to regulate these products, in the context of their existing regulatory systems and the evolving evidence base about the health impacts of their use (including over the long term). The need for further research in this area is urgent, so that all countries considering regulatory strategies in relation to e-cigarettes and other electronic nicotine-delivery systems can be informed by the best, most up to date, evidence (noting that regulatory responses may also need to evolve over time as the evidence base continues to develop).

## Conclusions

In the decade since the WHO FCTC was adopted, substantial progress in tobacco control research and policy action has been made, but the overall level of investment in tobacco control research remains miniscule contrasted with the need and the resources of the tobacco industry. Recent research indicates that noncommunicable diseases generally receive a tiny fraction of total Official Development Assistance, and a very small percentage of this funding is allocated to research. In 2007, a mere 2.3% of overall development assistance for health was dedicated to all noncommunicable diseases [40]. Most LMICs lack the basic capacity for any tobacco control research, and major disciplinary gaps in behavioral sciences, economics, and law impede progress in developing policy-relevant research. Tobacco control needs to learn urgently from HIV/AIDS, malaria, and nutrition about the value and power of bringing together leading philanthropic foundations, development agencies, national research bodies, and appropriate private sector interests in an alliance to address the massive needs discussed here.

There are simply no better opportunities in global health to prevent up to a billion deaths in this century.

## Abbreviations

FCTC: Framework convention on tobacco control; FDA: U.S. Food and Drug Administration; ITC: International Tobacco Control Project; LMICs: Low- and middle-income countries; NCI: National Cancer Institute; NIH: National Institutes of Health; TOBAC: (Fogarty International Center’s) International Tobacco and Health Research and Capacity Building program; WHO: World Health Organization.

## Competing interests

The authors declare that they have no competing interests.

## Authors’ contributions

DY and AP led the drafting of the manuscript, and all other authors contributed to the development and finalization of the manuscript. All authors read and approved the final manuscript.

## Authors’ note

The views expressed in this article are those of the authors and do not necessarily reflect the position or policy of the NIH, the Fogarty International Center, the WHO, or participants at the NIH-Fogarty workshop held in June 2013 on which some of the themes and conclusions in the article are based.
